# Memory B-cell derived donor-specific antibodies do not predict outcome in sensitized kidney transplant recipients: a retrospective single-center study

**DOI:** 10.3389/fimmu.2024.1360627

**Published:** 2024-04-05

**Authors:** Dania Altulea, Joost C. van den Born, Arjan Diepstra, Laura Bungener, Dagmar Terpstra, Bouke G. Hepkema, Rosa Lammerts, Peter Heeringa, Sebastiaan Heidt, Henny Otten, Leon Reteig, Gonca E. Karahan, Stefan P. Berger, Jan-Stephan Sanders

**Affiliations:** ^1^ Division of Nephrology, Department of Internal Medicine, University Medical Center Groningen, University of Groningen, Groningen, Netherlands; ^2^ Department of Pathology and Medical Biology, University of Groningen, University Medical Center Groningen, Groningen, Netherlands; ^3^ Transplantation Immunology, Department of Laboratory Medicine, University Medical Center Groningen, University of Groningen, Groningen, Netherlands; ^4^ Department of Immunology, Leiden University Medical Center, Leiden, Netherlands; ^5^ Center of Translational Immunology, University Medical Center Utrecht (UMC Utrecht), Utrecht, Netherlands

**Keywords:** memory B cells, DSA, polyclonal activation, IgG production, ABMR

## Abstract

**Background:**

Repeated exposure to sensitizing events can activate HLA-specific memory B cells, leading to the production of donor-specific memory B cell antibodies (DSAm) that pose a risk for antibody-mediated rejection (ABMR) in kidney transplant recipients (KTRs). This single-center retrospective study aimed to identify DSAm and assess their association with outcomes in a cohort of KTRs with pretransplant serum donor-specific antibodies (DSA).

**Methods:**

We polyclonally activated pretransplant peripheral blood mononuclear cells (PBMCs) from 60 KTRs in vitro, isolated and quantified IgG from the culture supernatant using ELISA, and analyzed the HLA antibodies of eluates with single antigen bead (SAB) assays, comparing them to the donor HLA typing for potential DSAm. Biopsies from 41 KTRs were evaluated for rejection based on BANFF 2019 criteria.

**Results:**

At transplantation, a total of 37 DSAm were detected in 26 of 60 patients (43%), of which 13 (35%) were found to be undetectable in serum. No significant association was found between pretransplant DSAm and ABMR (P=0.53). Similar results were observed in a Kaplan–Meier analysis for ABMR within the first year posttransplant (P=0.29). Additionally, MFI levels of DSAm showed no significant association with ABMR (P=0.28).

**Conclusion:**

This study suggests no significant association between DSAm and biopsy-proven clinical ABMR. Further prospective research is needed to determine whether assessing DSAm could enhance existing immunological risk assessment methods for monitoring KTRs, particularly in non-sensitized KTRs.

## Introduction

Formation of DSA remains a significant challenge for achieving long-term graft survival, contributing to both acute and chronic ABMR in transplant recipients. Sensitization to HLA antigens, leading to DSA development, can result from previous transplants, blood transfusions, pregnancies, or *de novo* occurrences post-transplantation, even with immunosuppressive therapy (1). This process involves the generation of long-lived plasma cells and HLA-specific memory B cells. Several studies have shown that HLA-specific memory B cells can still be detected against HLA antigens expressed on previous kidney allografts that failed many years prior, therefore suggesting their role in rejection and showing their long-term survival capacity (2, 3).

Historically, immunological risk assessment in solid organ transplantation relied on complement-dependent cytotoxicity crossmatch tests (CDC) and later incorporated more sensitive flow cytometry crossmatching assays (4). Currently, the evaluation of humoral immunity, driven by antigen-specific B cells, primarily involves detecting and characterizing HLA antibodies in recipient serum using solid-phase technology such as Luminex SAB assays (5, 6). Alongside antibody-focused risk assessment techniques, there has been an increasing interest in adaptive cellular memory screening, such as the detection of donor-reactive memory B and T cells. Few studies and a clinical trial (2, 3, 7, 8) have explored the roles of these cells in kidney transplant recipients (KTRs), particularly using ELISpot assays, which have shown promising results in predicting transplant outcomes in small groups of KTRs. However, due to the lack of result reproducibility, these assays have not yet been implemented in routine clinical settings.

In addition to the ELISpot, Wehmeier and colleagues developed another screening assay that uses SAB to detect DSAm by concentrating IgG antibodies from the activated B cells culture supernatant *in vitro* (2). Their pilot study indicated that DSAm correlated with adverse transplant outcomes, notably biopsy-proven ABMR (including subclinical cases) (2). Given the growing interest in the role of adaptive cellular memory, especially donor-reactive memory B cells, in kidney transplant outcomes, and the need to evaluate the reproducibility of these cell-based assays for clinical implementation, our study aimed to validate and expand upon the findings of Wehmeier and colleagues, examining the clinical implications of DSAm in a larger cohort of kidney transplant recipients with pretransplant DSAs. We focus on biopsy-proven clinical ABMR to assess its association with DSAm similarly to the previous study.

## Materials and methods

### Study design and patient population

This single-center retrospective study was performed within the PROCARE Consortium (9). Donors and recipients were HLA typed as part of the PROCARE study, and additional high-resolution typing was performed later in this study when needed to interpret the DSA status. The presence of DSA was determined by the SAB assay using the most recent, heat-inactivated serum samples before transplantation (except in cases of insufficient material for future clinical crossmatching, where earlier samples were used) (9–11). Transplants were performed following negative CDC crossmatch tests. Patients were selected based on positive pretransplant DSA presence and had received a transplant between 1995 and 2005 at the University Medical Center Groningen (UMCG). All materials were collected within the TransplantLines biobank. The Medical Ethical Committee of the UMCG approved the TransplantLines study protocol (METc 2014/077), and all study procedures were performed in line with the principles of the Declaration of Helsinki.

A total of 64 patients with pretransplant DSA were identified. Among these, two patients had undergone multiple transplants, and each was considered a separate case, while four patients were excluded due to the absence of PBMCs. PBMCs were isolated from heparinized blood using the standard density separation technique routinely performed at the transplant immunology laboratory at UMCG with Lymphoprep™ density gradient medium (STEMCELL Technologies Inc., USA). These isolated PBMCs were stored in liquid nitrogen until further use. The collection of patient materials (e.g., PBMCs and serum samples) was done 24-48 hours prior to transplantation except for a few cases with regards to the sera used for the SAB. In those cases, the most recent sample was taken instead. Further details regarding patient inclusion and other parameters are summarized in **Supplementary Figure S1**.

### Biopsy and rejection assessment

Kidney biopsies from 41 KTRs were reevaluated through blind scoring by a pathologist (AD) in accordance with the 2019 BANFF criteria for the purpose of this study to reflect the current acceptable criteria for defining rejection (12). When multiple biopsies were available for a single patient, the initial biopsy that was previously evaluated as having or suspected of having “rejection” according to the accepted standards at the time was selected for rescoring. The median time between transplantation and biopsy was 19 (10–69) days.

### Immunosuppression

Most patients (n=44) received induction therapy with either anti-thymocyte globulin (ATG; n=28), a monoclonal anti-interleukin-2 receptor antibody (anti-IL-2R; n=14), or muromonab-CD3 OKT3 (n=2). Most patients received a combination of triple immunosuppressive medications for maintenance including a calcineurin-inhibitor (e.g., cyclosporin (n=46) or tacrolimus (n=13)), corticosteroids (n=60), an anti-proliferative agent (e.g., mycophenolate mofetil (MMF; n=52), others (n=3). In this cohort, T cell mediated rejection was treated either with methylprednisolone or ATG in severe cases, and plasma exchange in patients with humoral rejection.

### *In vitro* polyclonal activation of B cells and processing of culture supernatants

PBMCs were thawed, washed, counted, and assessed for viability using Coulter cell counter (Beckman Coulter Life Sciences, USA) and NucleoCounter NC-200 (ChemoMetec, Allerod Denmark) respectively. Then, polyclonal activation of B cells within the PBMC population was performed following a previously published protocol (13). Briefly, thawed PBMCs were cultured at a concentration of 2 million cells/mL in 1 mL supplemented Iscove’s modified Dulbecco’s medium (IMDM; Lonza, Germany) in Costar® 24-well cell culture plates (Corning Life Sciences, USA) with a density of 2 million cells per well. At least, 4 wells per KTR containing 8 million PBMCs were cultured. The cells were then treated with 1mL “stimulation cocktail” consisting of 2.5 μg/mL Toll–like receptor 7/8 agonist resiquimod (R848; Sigma-Aldrich, USA) and 1000 IU/mL IL–2 (Proleukin® (aldesleukin); Clinigen Inc, USA). The plates were incubated for 10 days at 37°C with 5% CO_2_ in a standard cell culture incubator. On the 10^th^ day, the cell suspensions were collected into a round bottom 12mL tube for each patient. The tubes were then centrifuged for 7 minutes at 1700rpm, culture supernatants were collected, and stored at -20°C. The following day, neat culture supernatants were assessed by ELISA for total IgG concentration using an in-house developed sandwich ELISA as previously described (14).

### IgG concentration

Using the collected culture supernatant, IgG was isolated and concentrated using Amicon™ Pro Affinity concentration Kit and Amicon™ ProPurification Device (Millipore, USA) following the manufacturer’s instructions and a published protocol (13). In brief, 8-10mL of culture supernatant was added to a protein G containing resin in a purification column and incubated, then centrifuged to isolate the IgG. Subsequently, 50kD centrifugal filter units were added to the columns (Amicon Ultra – 0.5mL Centrifugal Filters; Millipore, Ireland), and IgG was eluted with the provided elution buffer. Finally, the concentrated IgG was collected from the 50kD filters by centrifugation. Overall, 29-34μL of eluates containing isolated IgG were obtained from approximately 8mL of starting neat supernatant volume. Concentrated IgG eluates were stored at -20°C until further use.

### HLA antibody detection in concentrated eluates of culture supernatants

HLA class I and class II antibody detection in the IgG eluates was performed using Lifecodes single antigen bead kits (LSA; Immucor Transplant Diagnostics, Stamford), by the transplant immunology laboratory at the UMCG following the manufacturer’s instructions. The data were analyzed with MATCH IT! Antibody v1.3.1 software provided by the company (Immucor, Stamford). The cutoff value used to define bead positivity followed the recommendations of the manufacturer, and the calculation of background corrected median fluorescence intensity (MFI) values (BCM) was done by the software. Memory B cell DSA was assigned when the mismatched allele was present in the donor. Additional high-resolution typing was performed when needed to interpret the DSA status. Only the results of a single pretransplant serum measurement were used for comparison with the SAB assay results from the IgG eluates. Following this, the presence of DSAm was determined by comparing the HLA specificities from the polyclonal B cell stimulations (referred to hereafter as HLAm) to the HLA type of the donor. The results were validated by a transplant immunologist (LB) at the UMCG following the initial analysis.

### Statistical analysis

Statistical analyses and data visualization were performed using SPSS (IBM® Corp, USA) v28 software and GraphPad Prism v9.0 software. Survival comparisons were performed using the Log-rank test and visualized with Kaplan Meier plots. Categorical data are presented as counts and percentages and were analyzed by Fisher’s exact test. For the parametric data, the mean and standard deviation were used for presentation, and the t-test was used for comparison between groups. For the nonparametric data, the median and interquartile range were used for presentation, and the Mann-Whitney U test was used for comparison between groups. For all statistical tests, a P value less than 0.05 was considered statistically significant.

## Results

### Patient population

In total, 60 KTRs with at least one pretransplant DSA were included. Patient and transplant characteristics are summarized in the first column of **Table 1**. The second and third columns of **Table 1** summarize the characteristics of these KTRs with regard to their DSAm status. The mean age of the patients was 45 years. Of these, 60% were female. Most patients (n=50; 83%) received a transplant from a deceased donor. With regards to sensitization, 49 KTRs (82%) had a known sensitizing event, whereas for the rest (n=11; 18%), the sensitizing event was not known (e.g., blood transfusions, unreported pregnancies, or miscarriages).

**Table 1:** Patient baseline characteristics.

Characteristics	Study Population (n = 60)	DSAm_pos_ pretransplant (n=26)	DSAm_neg_ pretransplant (n=34)
**Age (years)**	44.6 ± 13.4	43 ± 13.8	46.1 ± 13.4
Sex			
Female	36 (60%)	19 (73%)	17 (50%)
Male	24 (40%)	7 (27%)	17 (50%)
Kidney Disease			
Glomerulonephritis	21 (35%)	6 (23%)	15 (44%)
Cystic and Congenital Disease	15 (25%)	8 (31%)	7 (21%)
Vascular Kidney Disease	9 (15%)	6 (23%)	3 (9%)
Pyelonephritis and Interstitial nephritis	9 (15%)	3 (12%)	6 (18%)
Diabetic Kidney Disease	2 (3.3%)	1 (4%)	1 (3%)
Other	4 (6.7%)	2 (8%)	2 (6%)
Dialysis			
Hemodialysis	38 (63%)	17 (65%)	21 (62%)
Peritoneal dialysis	17 (28%)	6 (23%)	11 (32%)
None	5 (8.3%)	3 (12%)	2 (6%)
Dialysis Time (days)	1317 ± 1002	1034 ± 663	1533 ± 1002
Type of Donor			
Deceased	50 (83.3%)	21 (81%)	29 (85%)
Living	10 (16.7%)	5 (19%)	5 (15%)
**Donor Age (years)**	40.2 ± 16.2	40.3 ± 16.3	41 ± 16.2
**Cold Ischemia Time (minutes)**	1072.2 ± 548	1074 ± 558	1071 ± 548
Sensitizing events			
Pregnancy only	23 (38%)	12 (46%)	11 (32%)
Retransplant only	22 (37%)	8 (31%)	14 (42%)
Pregnancy and retransplant	4 (6.7%)	2 (8%)	2 (6%)
Not Known (unreported pregnancy, blood transfusion, etc.)	11 (18.3%)	4 (15%)	7 (21%)
Panel reactive antibodies (PRA)			
Pre-transplant PRA (%; mean ± SD)	19.1 ± 29.4	11.6 ± 30	24.7 ± 29.4
Highest PRA (%; mean ± SD)	35.3 ± 36.1	35.2 ± 36.1	35.3 ± 36.1
Induction Therapy			
Yes	44 (73%)	20 (77%)	24 (71%)
No	16 (27%)	6 (23%)	10 (29%)
HLA mismatches (HLA-A, -B, -DR)			
Total (mean ± SD; max 6)	2.9 ± 1.3	3.1 ± 1.1	2.6 ± 1.4
Class I (mean ± SD; max 4)	2 ± 1.1	2.3 ± 0.9	1.8 ± 1.1
Class II (mean ± SD; max 2)	0.9 ± 0.5	0.9 ± 0.4	0.8 ± 0.5

Values are n (%) or mean ± standard deviation.

### *In vitro* polyclonal activation of B cells

The *in vitro* stimulation assay was performed in all 60 KTRs using frozen, pretransplant PBMCs that were collected as part of the PROCARE study (15). A total IgG ELISA was performed to assess the success of the stimulation assay and determine the concentration of the IgG in the neat culture supernatants. The median IgG concentration in the supernatant was 8 μg/mL after normalization against a human IgG standard.

### Comparison of pretransplant DSA in serum and eluates

All patients had their serum assessed for HLA antibodies in the PROCARE 1 study (11), and both HLA mismatches and DSA were identified. In all patients, at least one or more pretransplant DSA were detected as per inclusion criteria. The assigned DSA along with the background corrected MFI (BCM) and HLA mismatches are summarized in **Supplementary Tables S1A** and **B** respectively. Subsequently, concentrated IgG eluates from the polyclonal B cell stimulations were also assessed for HLAm. 47 patients (78%) had detectable HLAm antibodies (data not shown), and 44 (73%) patients had HLAm specificities that were different from the serum HLA specificities mismatched to the current transplant (**Supplementary Table S2**). In the HLAm positive group, 15 patients (32%) had HLAm class I only, 13 (28%) had HLAm class II only, and 19 (40%) had both. **Table 2** summarizes the assigned DSAm with the respective BCM (rounded to 500). In some cases (n=4), the MFI of the DSAm was low (<500) but since these beads were considered as “positive” by the software, they were included. A total of 37 DSAm were found in 26 (43%) patients, of which, 15 patients had DSAm against HLA class I (58%), 10 (38%) were against HLA class II, and 1 (4%) was against HLA class I and II. Interestingly, 13 (35%) out of the 37 identified DSAm were unique to the *in vitro* assay and were not detected in the serum using the SAB assay (shown in **Table 2** in bold). Notably, no statistical difference was found between the MFI values of the DSAm not detected in the serum when compared to the MFI values of the DSAm that were also detectable in serum (P=0.08).

**Table 2:** DSAm specificities and respective background corrected MFI.

Patient ID	DSAm Specificity	DSAm Background corrected MFI
8	DPB1*17:01	**15000**
13	DRB3*02:02, DPB1*04:02	**200, 1000**
15	B62, Cw5	15000, 12000
18	B65	400
21	DQB1*04:01	9000
22	**A11, B27**	11000, 7000
23	DRB1*11:01, DPB1*02:01	12000, **900**
24	B38	19000
26	A11, **Cw3**	20000, **15000**
29	B62	11000
30	**B7**	**1300**
31	A1, DRB5*01:01	9000, **3000**
32	DRB1*11:01, DQB1*03:01	15000, 1500
34	B49, **B37**	1300, **11000**
37	A32, Cw7	4000, 500
40	A1	17000
45	DRB3*01:01DRB3*01:01	**200**
48	B27	6000
51	**A29, A11**	**2500, 350**
53	DRB1*01:01	900
54	B57, B37	18000, 1400
55	DRB1*11:01	**1500**
56	B44	11000
57	DQB1*03:01	**15000**
58	B56	2000
59	DRB5*01:01	2000

The DSAm unique to the *in vitro* assay are shown in bold.

### DSAm and the incidence of ABMR in biopsies

**Figure 1:**
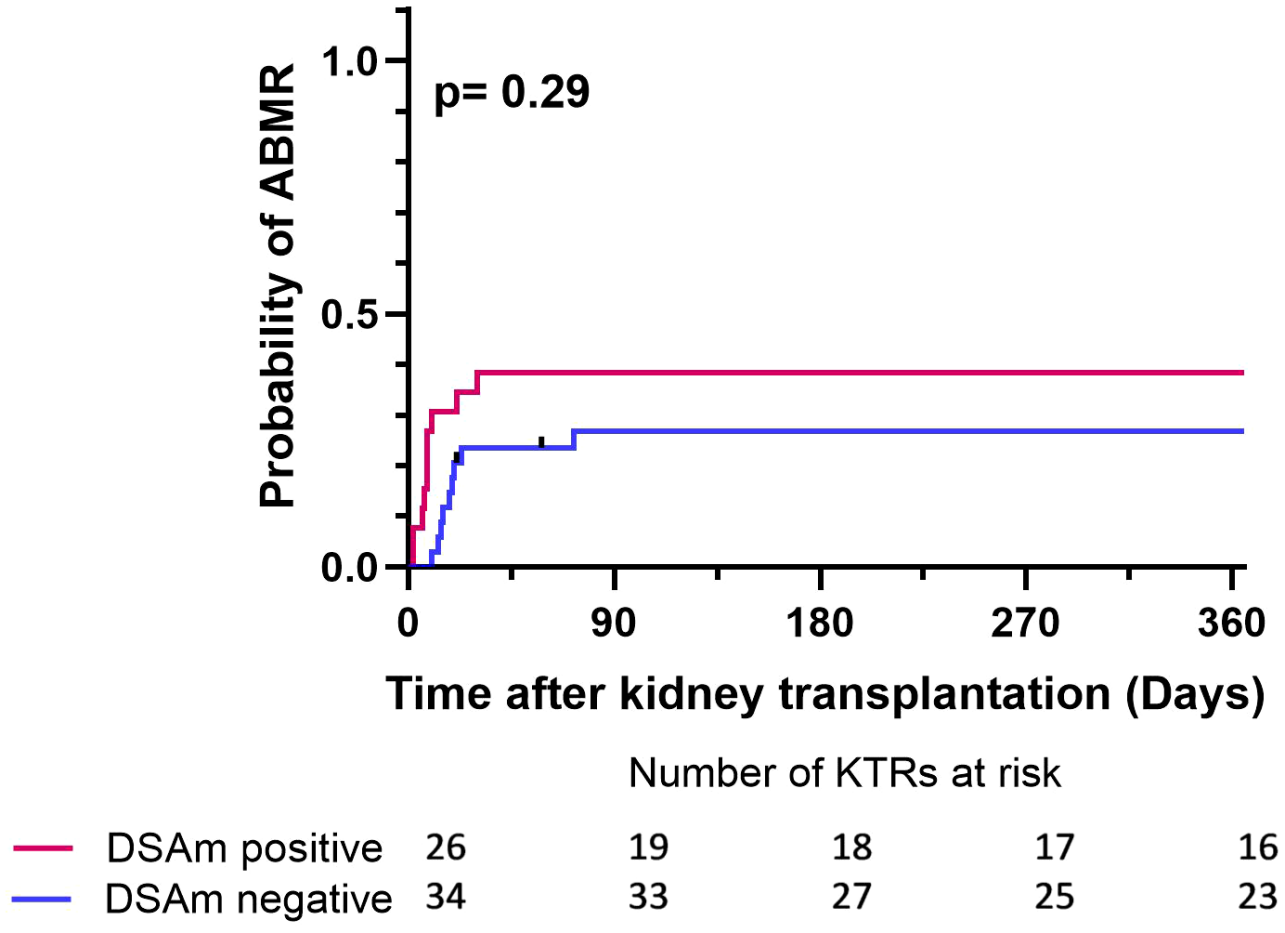
Death censored Kaplan-Meier survival analysis depicting the effect of DSAm on the incidence of ABMR with the number of patients at risk. P=0.29, probability of ABMR in the DSAm positive KTRs = 0.39, probability of ABMR in the DSAm negative KTRs = 0.32.

To correlate the transplant outcomes with the DSAm status, indication biopsies, graft failure, as well as follow-up data from one year and 10 years posttransplant were analyzed. In total, 41 out of the 60 KTRs underwent indication biopsies at the time of suspected kidney function decline. **Table 3** provides a summary of the biopsy results, divided between the DSAm positive and negative groups. In total, 22 out of the 60 patients (36%) had biopsies scored as ABMR; 11 patients (47%) in the DSAm positive group and 11 (40%) in the negative group (P=0.58). This lack of statistically significant association is further emphasized in **Figure 1** in which the relationship between the DSAm status and the occurrence of ABMR was assessed with a death-censored analysis in the first year posttransplant (P=0.29), and the probability of ABMR development in the DSAm negative and positive groups were 0.3 and 0.4 respectively. Finally, no association was found between the identified DSAm MFI levels and the occurrence of ABMR (P=0.28). With regards to TCMR, there was no significant difference in the incidence between the DSAm positive and negative groups (P=0.54).

**Table 3:** Biopsy analysis and follow-up data.

	DSAm_pos_ pretransplant (n=26)	DSAm_neg_ pretransplant (n=34)	p-value
**Number of indication biopsies assessed**	18 (69%)	23 (68%)	n.a
**Rejection (all)**	11 (40%)	16 (47%)	0.79
TCMR (total)	5 (19%)	10 (29%)	0.55
1A	2 (8%)	2 (6%)	0.99
1B	1 (4%)	5 (15%)	0.22
2A	1 (4%)	2 (6%)	0.99
2B	1 (4%)	1 (3%)	0.99
ABMR (total)	11 (42%)	11 (32%)	0.59
Acute	8 (30%)	8 (23%)	0.56
Chronic	3 (12%)	3 (8%)	0.99

TCMR, T-cell mediated rejection; ABMR, Antibody-mediated rejection. Chi-square and Fisher’s exact tests were used for the analysis. P<0.05. na, not applicable.

### Graft survival and follow-up data

**Figure 2:**
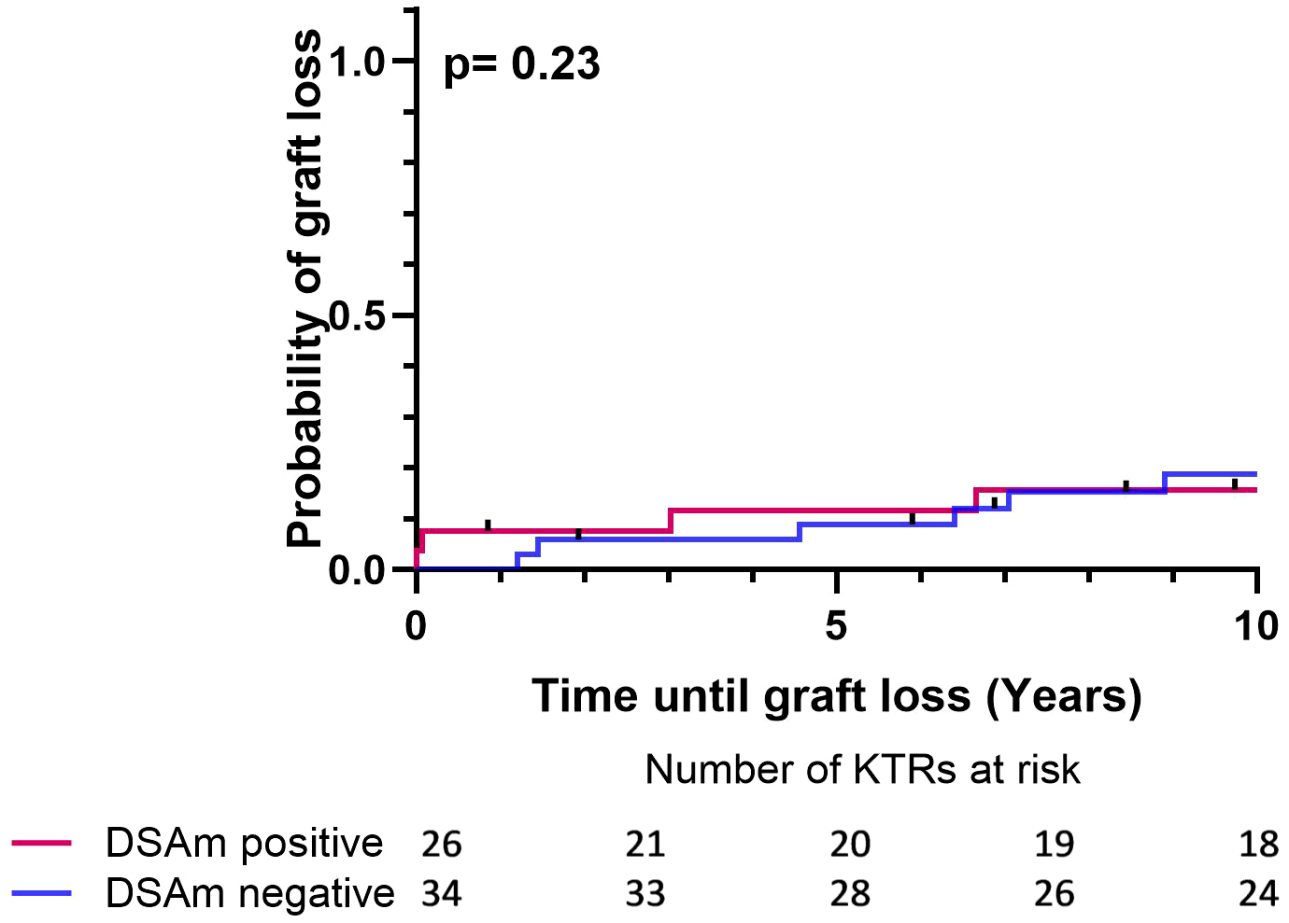
Death censored Kaplan-Meier survival analysis depicting the effect of DSAm on graft survival with the number of patients at risk. P=0.23, DSAm positive graft loss probability = 0.19, DSAm negative graft loss probability = 0.26.

Follow-up data (estimated glomerular filtration rate (eGFR) and proteinuria levels) for the first year posttransplant and 10 years after, as well as the transplant outcomes divided between DSAm positive and negative patients, are summarized in **Table 4**. In general, there were no differences between the groups in terms of the overall survival as well as the graft survival. Additionally, the clinical data did not show any differences in the proteinuria between the DSAm positive and negative groups, as these have stayed stable during the first follow-up year (P=0.91) and over 10 years posttransplant (P=0.91). Moreover, graft function as measured by eGFR was significantly better at one-year posttransplant (P=0.006) and at 10-year posttransplant as well (P=0.04) in patients who were DSAm positive compared to DSAm negative patients. In the first 10-year posttransplant, death-censored graft survival is presented in **Figure 2**. The analysis revealed no association between the presence of DSAm pretransplant and graft loss over 10 years (P=0.23), and the probability of graft loss in the DSAm negative and positive groups were 0.26 and 0.19 respectively.

**Table 4:** Graft outcomes and follow-up data.

	DSAm_pos_ pretransplant (n=26)	DSAm_neg_ pretransplant (n=34)	p-value
*Overall survival (up to 10 years posttransplant)*	23 (88%)	26 (76%)	0.32
*Graft survival (up to 10 years posttransplant)*	20 (77%)	24 (71%)	0.77
*Death censored graft loss (up to 10 years posttransplant)*	22 (85%)	28 (82%)	0.99
First year posttransplant			
eGFR data (n)	23	34	
eGFR (mL/min/1.73m^2^)	60 ± 18.3	47.2 ± 17.6	0.006
Proteinuria data (n)	22	33	
Proteinuria (g/24 h)	0.2 (0.1-0.5)	0.2 (0.1-0.4)	0.91
Ten years posttransplant			
eGFR data (n)	18	23	
eGFR (mL/min/1.73m^2^)	63.3 ± 26.7	46.6 ± 25.5	0.04
Proteinuria data (n)	17	19	
Proteinuria (g/24 h)	0.2 (0.1-0.55)	0.2 (0.1-0.52)	0.91

eGFR is presented as mean ± standard deviation. Proteinuria is presented as the median and (interquartile range). For the overall survival, graft survival, and death censored graft survival, Chi-square and Fisher’s exact tests were used for the analysis. For eGFR, a t-test was used for comparison, and for the proteinuria, the Mann-Whitney U test was used for comparison. P<0.05.

## Discussion

The primary findings of this study revealed that the detection of DSAm had no significant impact on the development of ABMR or rejection in general. Additionally, there was no correlation between DSAm and graft survival, and no association was observed between the levels of DSAm MFI and the occurrence of ABMR. As for the other clinical outcome measures, patients with DSAm showed significantly better graft function at one year and 10 years posttransplant. Interestingly, 13 unique DSAm specificities found in 11 patients were identified in the concentrated IgG eluates derived from polyclonally stimulated B cells that were not detected in serum, which we were able to attribute to a prior, patient-specific sensitizing event (i.e., pregnancy, retransplant, etc). However, the MFI values of this group of DSAm were not significantly different from the MFI values of DSAm that were also detectable in the serum. On the other hand, 11 DSA specificities that were previously detected in the serum were absent or undetectable in the concentrated IgG eluates from the *in vitro* assay in the DSAm positive group.

Despite significant advancements in immunological risk assessment technologies for detecting circulating anti-HLA antibodies, the contribution of the cellular memory compartment of humoral immunity (i.e., memory B cells) to the immunological risk of KTRs is still a subject of debate (16, 17). Various methodologies have been proposed to identify HLA-specific antibody-producing memory B cells, including flow cytometry analysis and sorting of B cells (18–20), as well as ELISpot assays for *in vitro* activated memory B cells (21, 22). As it stands, the main drawback of these assays is the lack of result reproducibility that makes it difficult to recommend them for widespread diagnostic use in clinics (17). However, we acknowledge that reproducibility could only be possible when all of the experimental variables have been accounted for, which in itself, can be difficult to achieve.

That said, and as suggested by evidence from previous studies, determining the impact of the memory B cell compartment is important for risk assessment, which is why attempts at increasing the sensitivities of these cell-based assays have been made. For example, B cell stimulation assays allow the production of IgG antibodies from memory B cells with higher concentrations, and their subsequent detection using the highly sensitive Luminex SAB could potentially be adapted to complement the existing transplant risk assessment strategies effectively.

The polyclonal activation of memory B cells for antibody production and subsequent detection using the SAB assays is an effective method for profiling the specificity of HLAm and it enables a direct comparison with serum HLA antibody profiles (2, 13). However, whether this approach will improve pretransplant risk stratification and thus impact clinical decision-making is still unknown. For instance, our study did not show a statistically significant relation between the presence of DSAm and ABMR within the first year after transplantation in a cohort of 60 immunologically high-risk KTRs unlike what was previously published by Wehmeier et al. (2) wherein the main conclusion was that patients with pretransplant DSA who tested positive for DSAm were more likely to experience biopsy-proven ABMR (including biopsies with subclinical ABMR) within the first year, compared to those pretransplant DSA-positive patients without DSAm. However, an important thing to note is that despite the lack of statistical association between the DSAm and ABMR, the DSAm positive group seems to have numerically more ABMR cases as seen in **Table 3** (n=11/26; 42%). This could suggest that DSAm does indeed have some contribution to ABMR, however, the magnitude of this contribution was not strong enough to reach statistical significance.

Similar to our findings, Wehmeier did not report any significant differences in long-term graft failure and function. We also observed similar findings for neat culture IgG concentrations (median of 8 μg/mL compared to 8.8 μg/mL), the percentage of patients with detectable HLAm (61% compared to 80%), and the percentage of patients with DSAm (43% compared to 45%). Therefore, we believe that the differing conclusions between the two studies are less likely due to technical assay differences, and more in part, due to differences in the patient groups and the type of and policies of the medical care provided. Additionally, this study used PROCARE’s HLA typing data complemented with next-generation sequencing (if needed) for the determination of DSA (10), while Wehmeier et al. used next-generation sequencing for all HLA typing and EDTA-treated serum for comparison to the DSAm. Apart from this, outcome variation likely arose from diverse patient cohorts and the unavailability of protocol biopsies for subclinical rejection assessment in our study (2). Specifically, Wehmeier et al. conducted protocol biopsies at different time points (3, 6, and 12 months after transplantation), and most of the biopsy-proven ABMR in their study resulted from these biopsies revealing subclinical rejection. In contrast, our cohort lacked protocol biopsies, and therefore, the assessment of subclinical rejection was not possible. Nevertheless, even though protocol biopsies were not conducted, nearly two-thirds of the patients underwent at least one biopsy where over half of the overall indication was due to a decline or delay in kidney function (n=29; 60%). Lastly, graft function (eGFR) was significantly better in the group positive for DSAm one-year posttransplant and at 10-year posttransplant (P=0.006, P=0.04 respectively), which was an unexpected finding and that could partly be explained as the result of differences in the baseline characteristics between the DSAm-positive vs. DSAm-negative groups. At baseline the groups were unbalanced for age (3 year younger at baseline for DSAm-positive group), sex (more female in the DSAm-positive group), and primary kidney disease (glomerulonephritis being more prevalent in the DSAm negative group compared to the DSAm positive).

This study has several limitations. Its retrospective design led to the inclusion of patients based on the availability of a sufficient number of PBMC vials to conduct the polyclonal activation assay, therefore potentially introducing a selection bias. Further, the presence of pretransplant DSA as a prerequisite to the inclusion could be considered a confounding variable as these serum DSAs could have diluted the effect DSAm might have on the outcome. Moreover, the reliance on indication biopsies for diagnosing rejection may have resulted in overlooking patients with subclinical rejection, which could have been influenced by the presence of DSAm pretransplant as demonstrated by Wehmeier et al. (2) Additionally, the study did not assess whether the identified pretransplant DSAm were also detectable posttransplant, as the primary focus was on the risk assessment of pretransplant DSAm. Finally, since all of the included KTRs in the study were transplanted during a time period where DSA assessment with SAB was not performed at the time of transplantation, it is highly likely that the presence of DSA pretransplant was not known or partially known for most of these patients, and therefore, factors such as the immunosuppressive medication (i.e., the use of heterogenous immunosuppressive regimens) might not have been fully taken into account which could have influenced the occurrence of ABMR. Despite these limitations, the study included 60 immunological high-risk patients selected retrospectively from a large, well-characterized cohort, providing high-quality characterization data and long-term follow-up.

To summarize, this retrospective single-center study has shown that evaluating DSAm before transplantation did not improve the ABMR risk assessment in a cohort of 60 KTRs with pretransplant DSA, as no clear associations between the presence of pretransplant DSAm and the incidence of ABMR were observed. Future prospective studies focusing on the role of DSAm in high-risk KTRs are necessary to gain further insights into its potential advantages in predicting transplant outcomes. For example, the value of this assay could be potentially more impactful in groups of sensitized patients without pretransplant serum DSA, or patients with pretransplant HLA antibodies without a (known) sensitizing event. These groups might be more likely to benefit from an expanded risk assessment protocol and should therefore be also included in future studies. However, as it stands, this assay is still in its “research stage” and is not yet ready to enter routine practice.

## Data availability statement

The original contributions presented in the study are included in the article. Further inquiries can be directed to the corresponding author.

## Ethics statement

The medical ethical committee of the University Medical Center Groningen approved the study protocol (METc 2014/077). The human samples used in this study were collected from study participants after they provided informed written consent.

## Author contributions

DA: Writing – review & editing, Writing – original draft, Methodology, Investigation, Formal analysis, Conceptualization. JV: Writing – review & editing, Formal analysis, Data curation, Conceptualization. AD: Writing – review & editing, Validation, Resources, Methodology. LB: Writing – review & editing, Validation, Formal analysis, Data curation. DT: Writing – review & editing, Investigation, Data curation. BH: Writing – review & editing, Validation, Supervision, Formal analysis, Data curation. RL: Writing – review & editing, Validation, Supervision, Formal analysis, Data curation. PH: Writing – review & editing, Validation, Supervision, Formal analysis, Conceptualization. SH: Writing – review & editing, Validation, Formal analysis, Conceptualization. HO: Writing – review & editing, Validation, Formal analysis, Data curation. LR: Writing – review & editing, Data curation. GK: Conceptualization, Writing – review & editing. SB: Validation, Supervision, Funding acquisition, Conceptualization, Writing – review & editing. J-SS: Validation, Supervision, Funding acquisition, Conceptualization, Writing – review & editing.

## Supplementary material

Supplementary Figure 1 A Flowchart showing the initial selection and exclusion and the number of patients who had received induction therapy (with ATG; n=28, anti-IL-2R; n=14, or muromonab-CD3 OKT3; n=2) and had indication biopsies taken. Created with BioRender.com.
