# Supplementary figures and images for: Memory B-cell derived donor-specific antibodies do not predict outcome in sensitized kidney transplant recipients: a retrospective single-center study
Source: Front Immunol. 2024 Apr 5;15:1360627. doi: 10.3389/fimmu.2024.1360627 (PMC11026632; doi:10.3389/fimmu.2024.1360627)

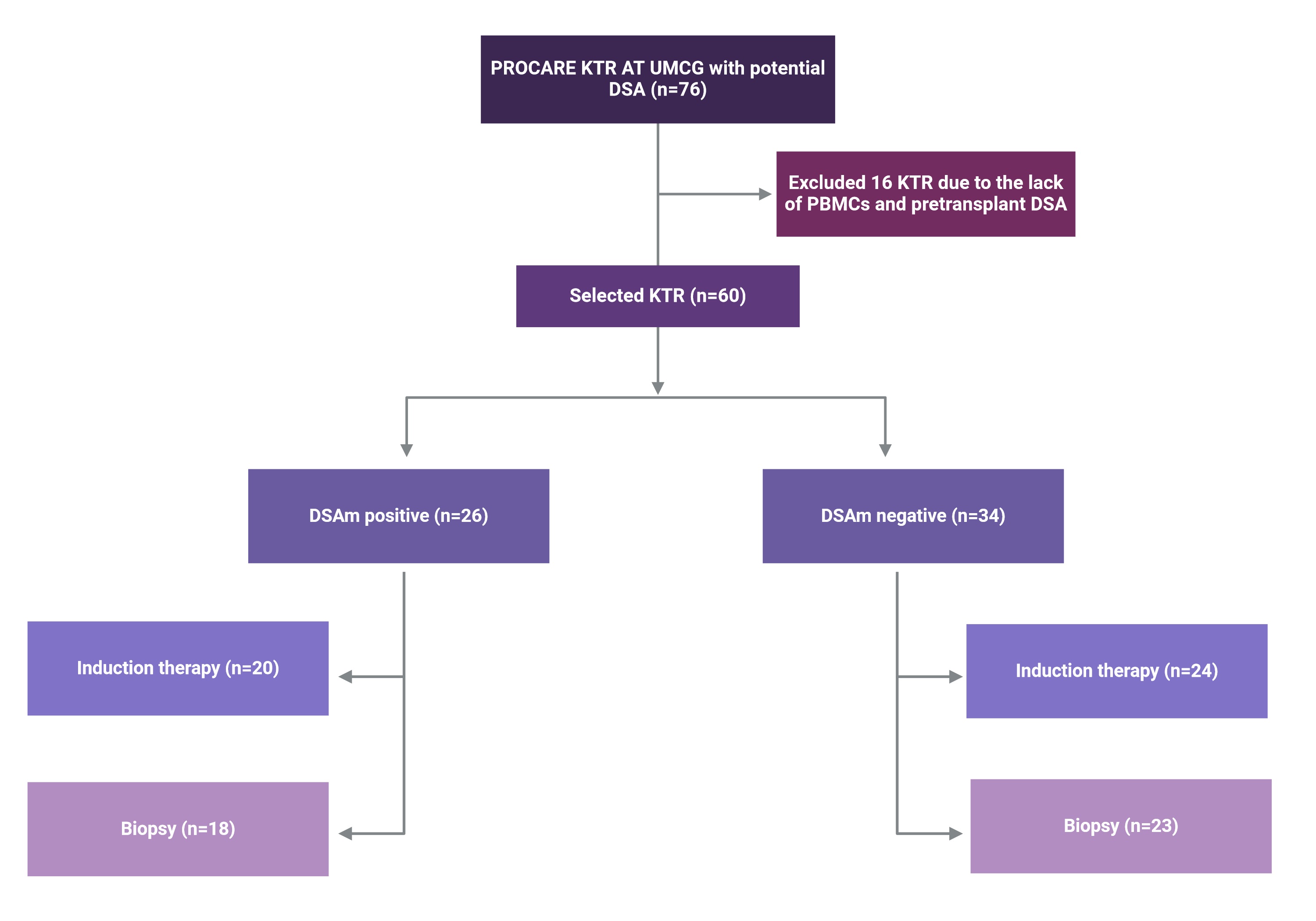

Supplement: Supplementary Figure 1 — A Flowchart showing the initial selection and exclusion and the number of patients who had received induction therapy (with ATG; n=28, anti-IL-2R; n=14, or muromonab-CD3 OKT3; n=2) and had indication biopsies taken. Created with BioRender.com. [file Image_1.jpg]
