# Supplementary material for: Memory B-cell derived donor-specific antibodies do not predict outcome in sensitized kidney transplant recipients: a retrospective single-center study
Source: Front Immunol. 2024 Apr 5;15:1360627. doi: 10.3389/fimmu.2024.1360627 (PMC11026632; doi:10.3389/fimmu.2024.1360627)
Supplement: Supplementary file 2 [file Table_1.docx]

***Table S1-A:*** *Summary of the assigned pretransplant donor-specific HLA antibodies (DSA) with the background corrected MFI (BCM) rounded off at 500. The DSA that were absent or undetectable in the IgG eluates from the in vitro assay in the DSAm positive group are shown in bold.*

| **Patient ID** | **Specificity** | **BCM** |
| --- | --- | --- |
| 1 | B35 | 4500 |
|  | DR12 | 2000 |
| 2 | DQ5 | 3500 |
|  | DR1 | 14000 |
| 3 | DQ4 | 1000 |
| 4 | DR4 | 2000 |
| 5 | A1 | 1500 |
| 6 | DQ6 | 2500 |
| 7 | B44 | 4000 |
|  | DQ4 | 9500 |
| 8 | **A33** | **2500** |
| 9 | DQ6 | 14000 |
| 10 | B44 | 3500 |
| 11 | DQ6 | 3500 |
| 12 | DR14 | 1500 |
|  | DQ5 | 2000 |
| 13 | **B18** | **1000** |
| 14 | DR12 | 3500 |
| 15 | B62 | 3500 |
|  | Cw5 | 10000 |
| 16 | B58 | 2500 |
| 17 | A11 | 13000 |
| 18 | B65 | 2000 |
|  | **Cw8** | **1500** |
| 19 | DR4 | 1500 |
| 20 | DR13 | 1000 |
| 21 | **DR8** | **4500** |
|  | DQ4 | 19000 |
| 22 | A11 | 9000 |
|  | B27 | 13000 |
|  | **DR1** | **3000** |
| 23 | DRB11 | 3500 |
| 24 | B38 | 4000 |
| 25 | A24 | 1000 |
| 26 | A11 | 3000 |
| 27 | A2 | 1500 |
| 28 | A11 | 6000 |
| 29 | B62 | 6000 |
| 30 | **A2** | **2000** |
| 31 | A1 | 2000 |
| 32 | **B18** | **1500** |
|  | DQ7 | 3500 |
|  | DR11 | 17000 |
| 33 | DQ2 | 3000 |
| 34 | B49 | 1000 |
| 35 | A1 | 2500 |
| 36 | B35 | 1000 |
| 37 | A32 | 3000 |
|  | Cw7 | 2000 |
| 38 | DQ4 | 2000 |
| 39 | DR11 | 1500 |
| 40 | A1 | 1000 |
| 41 | DQ7 | 1000 |
| 42 | B44 | 1000 |
| 43 | A24 | 1500 |
| 44 | A*68:02 | 500 |
|  | A2 | 2500 |
| 45 | **A24** | **2000** |
| 46 | DR11 | 1000 |
| 47 | DQ5 | 2000 |
| 48 | B27 | 2500 |
| 49 | DQ6 | 2500 |
| 50 | DQ8 | 3500 |
| 51 | **B*51:01** | **1000** |
| 52 | B62 | 4000 |
| 53 | **B7** | **500** |
|  | DR1 | 4000 |
| 54 | B37 | 5000 |
|  | B57 | 2000 |
| 55 | **DQ7** | **1500** |
| 56 | B44 | 4000 |
| 57 | DR4 | 500 |
| 58 | B56 | 1500 |
| 59 | DR15 | 6500 |
| 60 | B13 | 2000 |

***Table S1-B:*** *Summary of HLA mismatches based on typing known at transplant (HLA-A, -B, -C, -DR, -DQ). In case antibodies were found against loci that were not typed at the time of transplant, additional typing using NGS was performed to determine if these were DSA (e.g., DQB1, DPA1, DPB1, DRB345 alleles). These additional mismatches are added in italic this table.*

| **Patient ID** | **Mismatches** |
| --- | --- |
| 1 | B8 B35 Cw4 DR12 |
| 2 | B44 DR1 DQ5 |
| 3 | B44 Bw4 DR8 DQ4 |
| 4 | A2 B62 Cw3 DR4 DR53 DQ3 |
| 5 | A1 B8 B39 Bw6 Cw12 |
| 6 | DR15 DR51 DQ6 |
| 7 | A3 B44 B62 Cw3 Cw5 DR4 DR8 DR53 DQ4 |
| 8 | A33 B65 B61 Cw8 Cw15 DR18 DQ4 *DPB1*17:01, DPA1*02:01* |
| 9 | A11 B38 B60 Bw4 Cw3 Cw12 DR15 DR51 DQ6 |
| 10 | B44 Bw4 Cw5 |
| 11 | A11 B7 Cw7 DR15 DR51 DQ6 |
| 12 | A29 B44 DR14 DR52 DQ5 |
| 13 | B18 *DRB3*02:02 DPB1*04:02* |
| 14 | A24 A33 B75 B77 Bw4 DR12 DR52 |
| 15 | A2 B62 Cw10 Cw5 DQ8 DQ4 |
| 16 | A30 A31 B58 B18 Cw5 Cw7 DR1 DR3 DQ5 |
| 17 | A11 |
| 18 | A30 B65 B60 Cw3 Cw8 DR8 DQ4 |
| 19 | DR4 DQ3 |
| 20 | B27 Bw4 Cw2 DR13 |
| 21 | B51 DR8 DQ4 |
| 22 | A11 B27 Cw2 Cw3 DR1 DQ1 |
| 23 | DR11 *DPB1*02:01* |
| 24 | A26 B38 Bw4 |
| 25 | A24 B39 DR1 DQ1 |
| 26 | A11 B55 Cw3 DR14 |
| 27 | A1 A2 Cw6 DR15 DR51 DR53 |
| 28 | A11 |
| 29 | A3 B62 Cw1 DR9 DR53 DQ9 |
| 30 | A2 B7 Cw3 DR7 |
| 31 | A1 B62 B49 Cw3 *DRB5*01:01* |
| 32 | B18 DR11 DQ7 |
| 33 | B51 DR103 DR53 DQ2 |
| 34 | A1 B49 B37 DR7 DQ2 DQ4 |
| 35 | A1 B8 DR8 |
| 36 | B35 Cw3 Cw4 |
| 37 | A32 B8 Cw7 DR3 DQ2 |
| 38 | A25 A32 DR8 DQ7 DQ4 |
| 39 | B39 DR11 DQ7 |
| 40 | A1 Cw6 DR1 DQ9 DQ5 |
| 41 | B60 DQ7 DQ8 |
| 42 | A24 B44 Bw4 |
| 43 | A24 A28 Cw1 Cw2 DR1 DQ5 |
| 44 | A2 B7 B39 Cw7 DR8 DQ4 |
| 45 | A1 A24 B8 DR17 DR52 DQ2 |
| 46 | A3 B18 DR11 DR52 DQ3 |
| 47 | DR14 DQ5 |
| 48 | A2 B27 Bw4 Cw1 DR1 DQ5 |
| 49 | A2 A23 B44 Cw5 DR13 DQ6 |
| 50 | DQ8 |
| 51 | A11 A29 B51 Cw15 DR4 DR53 |
| 52 | B62 DR13 DR52 |
| 53 | A1 A3 B7 B8 Cw7 DR1 DR4 DQ5 DQ3 |
| 54 | A1 B57 B37 Bw4 Cw6 |
| 55 | A3 B44 B49 Cw5 DR11 DR52 DQ7 |
| 56 | A26 A11 B44 B62 Cw3 Cw4 DR4 DR53 DQ8 |
| 57 | A3 B7 B44 Bw4 Cw5 DR4 DQ3 *DQB1*03:01* |
| 58 | A3 B56 Bw6 DR1 DQ1 |
| 59 | A26 B45 Cw6 DR15 DR51 |
| 60 | A2 A31 B13 B39 Cw6 DR13 DR51 DR52 DQ6 |

***Table S2:*** *Summary of memory derived HLA antibodies (HLAm) against specificities not mismatched with the transplant as detected by the SAB assay. Patients with no detectable HLAm or those with HLAm specificities that were also mismatched in the serum were not included in this table.*

| **Patient ID** | **HLAm against specificities not mismatched in the serum** |
| --- | --- |
| 1 | DR51 DR9 DR103 DR10 DR1 |
| 2 | B56 DP3 DP5 DP1 DP6 DP13 DP9 DP19 DP14 DP11 DP17 |
| 3 | A1 A23 A24 A2403 B60 DR103 DR4 DR13 DR11 |
| 5 | A11 A66 A43 A3 |
| 6 | A68 A66 A34 A33 A25 A2 A26 A69 A203 A1 A43 A29 A74 A32 A31 B57 B58 B18 B3901 B38 B64 B65 B8 B63 B71 B73 B77 DR53 DR14 DQ8 DQ7 DQ4 DQ9 DQ2 |
| 8 | A32 B76 B44 B82 B45 B49 B52 B59 DPA2 DPA4 DP11 DP19 DP17 DP13 DP14 DP9 DP28 DP15 |
| 9 | DR9 DR7 DQ8 DQ9 DQ7 DQ4 |
| 12 | DR4 |
| 13 | A24 A23 A66 A1 A2403 A32 A25 A68 A2 A203 A69 Bw4 B76 B44 B82 B2708 B45 B7 B703 B81 B60 B27 B62 B47 B49 B63 B61 B71 B77 B53 B72 B52 B50 B13 B57 B75 B58 B35 B56 B59 B48 B38 B73 B51 B37 B78 B46 Cw17 Cw2 DR11 DR52 DQ8 DQ7 DQ9 DP4 DP28 DP2 DP18 |
| 15 | A2403 A24 A23 A32 A29 B13 B2708 B76 B75 B77 B57 B27 B63 B38 B58 B53 B60 B73 B61 B49 B59 B47 B46 B54 B67 B52 B48 B51 Cw6 Cw18 Cw4 Cw15 Cw17 Cw2 DR51 DR1 DR10 DR9 DR103 DR15 DR16 DR1403 DR18 DR12 DR11 DR13 DR17 DQ5 DQ6 DQ2 DP4 DP18 DP2 DP28 DP15 |
| 16 | DQ2 |
| 18 | A68 A34 A33 A69 B8 B59 B67 B2708 B27 B42 B54 B7 B81 B3901 B55 B73 B37 B38 B56 B18 B82 DR52 |
| 19 | A68 B7 B55 B42 B81 B67 B2708 B54 B703 B56 Cw8 Cw5 Cw7 DR1404 DR14 DR15 |
| 21 | DR13 DR4 DR11 DR103 DR12 DQ6 DQ5 DQ8 DQ9 DQ7 |
| 22 | A66 A25 A29 A26 A30 A34 A31 A33 A43 A68 A2403 A203 A2 A69 A36 B2708 B60 B61 B57 B47 B703 B7 B13 B58 B81 DR52 DR11 DR10 DR9 DR7 DQ7 |
| 23 | DR14 DR13 DR18 DR17 DR1403 DR7 DQ8 DQ9 DP6 DP3 DP18 DP9 DP14 DP17 DP4 DP2 DP28 |
| 24 | A32 A2403 A25 A24 A23 A1 B44 B63 B27 B49 B57 B76 B37 B47 B58 B77 B53 B59 B82 B51 B13 B45 B52 B8 |
| 25 | A66 A29 B703 B60 B7 B2708 B81 B61 B27 B47 B13 B48 B63 |
| 26 | A25 A66 A68 A26 A32 A43 A2403 A34 A33 A31 A29 A30 A2 A24 A23 A36 A69 A203 A74 B63 B49 B52 B51 B59 B77 B38 B73 B53 B57 B58 B46 B8 B42 B41 B13 B78 B50 B44 Cw14 Cw1 Cw10 Cw9 Cw12 Cw8 Cw16 Cw7 DR15 DR51 DR16 DR10 DR12 DQ6 |
| 27 | A3 Bw6 B76 B72 B62 B50 B49 B52 B71 B75 B56 B35 B703 B7 B81 B8 B53 B77 B78 B45 B63 B54 B44 B2708 B64 B3901 B67 B61 B82 B55 B42 B41 B60 B18 B65 B46 B59 B51 B48 B37 Cw10 Cw9 |
| 28 | A203 A2 A32 A2403 A25 A23 A24 A33 A69 A68 Bw4 B63 B77 B49 B51 B53 B57 B71 B58 B44 B52 B78 B38 B27 B59 B75 B62 B37 B13 B72 B56 B47 B35 B50 B46 B18 B82 Cw10 Cw9 DR52 |
| 29 | A32 A2403 A25 A23 A24 Bw4 B49 B50 B63 B53 B56 B72 B71 B52 B77 B38 B35 B59 B75 B51 B57 B37 B58 B27 B44 B47 B13 B78 B67 B41 B3901 B46 B60 B54 DR1 |
| 30 | A24 A23 A2403 B44 B13 B45 B49 B50 B47 B60 B61 B41 B703 B81 B67 DR103 DR1 DR9 DR51 DR10 DR11 DQ6 DQ5 DQ4 DPw6 DPw3 DPw4 DPw2 |
| 31 | A2 A203 A11 A3 A74 A32 A31 A30 A43 A36 A25 A66 A68 A33 A29 A34 A80 A26 A69 B57 B58 B63 B2708 B60 B27 B61 B13 B47 B703 B48 B44 B76 B45 B73 B81 Cw17 Cw4 Cw18 Cw6 DR13 DR11 DR14 DR18 DR1403 DR17 DR8 DR4 DR103 DR12 DR1404 DR51 DR16 DR52 DP18 |
| 32 | DR8 DR13 |
| 33 | A2 A203 A66 A69 A68 A3 A34 A33 A31 A29 A74 A11 A30 B2708 B7 B27 B60 B703 B81 B61 B73 B47 B67 B13 B55 B42 B56 B54 B72 B41 B48 B62 B49 B50 B3901 B38 B37 B44 B77 Cw2 |
| 34 | A2 A203 A68 A69 A66 A25 A34 A33 A26 A2403 A24 A32 A23 A43 A29 A74 A31 A30 A80 A36 B8 B81 B51 B703 B59 B7 B60 B78 B42 B57 B58 B54 B3901 B38 B73 B53 B41 B63 B52 B61 B67 B2708 B18 B35 B48 B64 B55 B27 B13 B65 B56 B77 B47 B71 B75 B46 Cw7 Cw2 DR15 DR53 DR13 DR4 DR16 |
| 37 | A203 A2 A68 A69 A34 A66 A43 A74 A26 A29 A25 A2403 A33 A31 B57 B73 B58 Cw17 |
| 38 | B75 B71 |
| 40 | A23 A24 A80 B76 B44 B82 B45 B49 B13 B60 B50 B61 B47 B41 B53 B72 B35 B18 B51 |
| 41 | DR11 DR14 DR1404 DR8 DR13 DR18 DR17 DR1403 DR12 DR52 |
| 43 | B7 B703 B81 |
| 44 | A33 A68 B63 B71 B53 B35 B52 B78 B75 B56 B77 B51 B49 B64 B18 B50 B54 B46 B8 B65 B62 B72 B59 B58 B57 B55 B42 B3901 B703 B38 Cw10 Cw9 DQ2 DP11 DP15 DR53 |
| 45 | A36 A2 A3 A32 A74 A203 B57 B58 |
| 47 | A1 A24 B76 DR7 DR9 |
| 48 | B2708 B60 B61 B703 B7 B13 B81 B48 B47 |
| 50 | DR11 DR14 DR13 DR8 DR17 DR18 DR12 |
| 51 | A203 A2 A69 A68 A2403 B7 B703 B2708 B81 B60 B27 B61 B67 B13 B42 |
| 52 | B2708 B82 B67 B42 B55 B81 B56 B7 B54 B57 B58 B63 B37 |
| 53 | DR15 DR16 DR103 DR7 DR51 |
| 54 | A2 A203 A68 A66 A34 A69 A33 A25 A26 A11 A2403 A29 A31 A43 A30 A36 A32 A74 B8 B7 B2708 B703 B81 B60 B27 B58 B42 B61 B47 B73 B41 B13 B59 B48 B67 B49 B63 B44 B50 B45 B55 B54 Cw2 |
| 56 | B45 B76 B13 B50 B49 B47 B60 B41 B61 B82 |
| 57 | DQ7 DQ8 DQ9 |
| 58 | A66 B2708 B60 B8 B61 B81 B703 B7 B27 B73 B13 B47 B59 B48 B77 B75 B67 B3901 B53 B35 B41 B42 B78 B50 B71 B52 B46 B49 B38 B18 B72 B51 B55 B54 Cw2 |

***Figure S1*** *A Flowchart showing the initial selection and exclusion and the number of patients who had received induction therapy (with ATG; n=28, anti-IL-2R; n=14, or muromonab-CD3 OKT3; n=2) and had indication biopsies taken. Created with BioRender.com.*
